# Bioinformatics Analysis of Potential Therapeutic Targets and Prognostic Biomarkers amid CXC Chemokines in Ovarian Carcinoma Microenvironment

**DOI:** 10.1155/2021/8859554

**Published:** 2021-01-28

**Authors:** Yunfeng Jin, Qiwang Lin, He Fei, Lujie Xue, Li Li, Qinghua Xi, Hua Jiang

**Affiliations:** ^1^Department of Gynecology, Obstetrics and Gynecology Hospital, Fudan University, Shanghai, China; ^2^Department of Obstetrics and Gynecology, Affiliated Hospital of Nantong University, Nantong, Jiangsu Province, China

## Abstract

**Background:**

Ovarian cancer (OC) is one of the leading lethal gynecologic cancers of women around the world. More than 70% of patients are diagnosed with stage III or IV with poor outcome. This is partly because of lacking early effective screening techniques and potential biomarkers of OC. CXC chemokines in tumor microenvironment (TME) and their interaction with relative receptors can excite the downstream signaling pathways to influence tumor progression. However, the role of CXC chemokines in OC has not been identified.

**Methods:**

ONCOMINE, GEPIA, Kaplan–Meier plotter, cBioPortal, TIMER, Metascape, and LinkedOmics were applied in our study.

**Results:**

The transcriptional levels of CXCL1/8/9/10/11/12/13/14/16/17 were significantly elevated while CXCL3 was obviously reduced in OC vs normal ovarian tissue. CXCL8/9/11/13 were correlated with clinic pathological stage. Patients with low expression of CXCL8/9/11/13 were associated with better prognosis. We also found that CXCL3 and CXC12 could be used as potential prognostic markers of OC through Kaplan–Meier plotter. Patients with high expression of CXCL3/12 had a significantly better prognosis. Their functions focus on locomotion, signaling, response to stimulus, undergoing the process of multiorganism, immune system, biological regulation, etc. The differentiated CXC chemokines mainly participate in cytokine-cytokine receptor interaction, chemokine signaling pathway, IL-17 signaling pathway, and toll-like receptor signaling pathway. Our results showed that CXC chemokines were highly correlated with infiltration of immune cells. The kinase targets of differentially expressed CXC chemokines are mainly in ATM, LYN, LCK, PLK1, FYN, CDK2, and ATR.

**Conclusions:**

Our results may provide a new insight for selecting precision biomarkers of targeted therapy of OC.

## 1. Introduction

Ovarian cancer is the most leading lethal gynecological cancer around the world. Patients with epithelial ovarian cancer (EOC), the most common pathological type of OC, are always diagnosed at late stages with poor five-year survival rate (FIGO stage III (51%) or IV (29%)) [1, 2]. Actually, cytoreductive/primary debulking surgery (PDS) followed by combined chemotherapy is an effective way to deal with early stage (stages I-IIA) patients, with a 5-year survival rate at around 90%. However, more than 70% of patients are diagnosed with advanced stages III-IV with poor outcome [3]. CA125 is still utilized as a traditional marker to diagnose and prognose this disease. Although targeted therapies, immunotherapies, and other combination therapies have showed their merits, it is far more sufficient to find more biomarkers and therapeutic targets to improve our early diagnostic standard and ameliorate the prognosis of OC patients.

Intricate cross-talk between cancer cells and other cells in TME exerts potential influence in neoplasia and tumor progression. Cytokines secreted by cancer cells and other TME cells exert significant influence on this orchestrated interaction. Chemokines (chemotactic cytokines), comprising pairs of ligands and their associated receptors, are a superfamily of low molecular weight cytokines. On the basis of their structure, chemokines are classified as C, CC, CXC, or CX3C, where *X* represents a nonconserved amino acid substitution [4]. Nearly 50 cytokines of human are divided into two functional groups: inflammatory chemokines and homeostatic chemokines. The primary function of chemokines is mediating cell trafficking and recruiting proinflammatory cells to the sites of inflammation [5]. Chemokines affect tumor progression via several aspects, including angiogenesis, proliferation, migration, invasion, and metastasis [6–8]. While chemokines may play a dual role in tumor development, some may favor tumor growth while some may favor antitumor immunity [5]. As their intriguing roles in tumors, chemokines represent an area of intense interest and study.

Similar to other tumor types, chronic inflammation is an important condition for ovarian cancer progression. The CXC group works as a key mediator in inflammation of OC. They are responsible for the recruitment and activation of immune cells in the inflammatory milieu; in addition, they mediate pro- and antiangiogenic effects. Chemokines are gaining importance in the field of ovarian cancer for being angiostatic and angiogenic in function [9]. Although several potential roles of chemokines in OC have been investigated, identifying precision therapy targets remains destitute. Recently, bioinformatics analysis of certain gene family in malignant tumors based on public databases has been emerging for us to delve into more practical information which may be applied in clinic therapies in the future [10–12]. Herein, in our study, we took advantages of public databases to comprehensively analyze the expression CXC family members in OC and further to evaluate their diagnostic and prognostic value to find more appropriate biomarkers applied in clinic.

## 2. Materials and Methods

### 2.1. ONCOMINE Analysis

ONCOMINE (http://www.oncomine.org) dataset is an online web-based cancer microarray database [13]. The mRNA expression of CXC chemokines in ovarian cancer was compared with normal controls. In our study, the cutoff of *p* value and fold change were set as 0.01 and 2, respectively. Student's *t*-test was used to analyze the differential expression of CXC in OC.

### 2.2. GEPIA Dataset

GEPIA (http://gepia.cancer-pku.cn) is an interactive web server applied in analyzing the mRNA sequencing data based on 9763 tumors and 8587 normal samples in the *Cancer* Genome Atlas (TCGA) and Genotype-Tissue Expression dataset project (GTEx). GEPIA provides customizable functions such as differential expression analysis, profiling according to cancer types and pathological stages, survival analysis, similar gene detection, and dimensionality reduction analysis [14]. In this study, we delved into a differential mRNA expression analysis of tumor and normal tissues, pathological stage analysis of CXC in OC via the module “Single Gene Analysis” of GEPIA.

### 2.3. The Kaplan–Meier Plotter Analysis

Kaplan–Meier plotter (http://www.kmplot.com) is an online database, including microarray gene expression data and survival information from Gene Expression Omnibus, TCGA, and the *Cancer* Biomedical information of 2190 OC patients [15]. OC patients were divided into two groups by median expression (high vs low) to analyze overall survival (OS), progression-free survival (FPS), and postprogression survival (PPS) through Kaplan–Meier plotter, with the hazard ratio (HR) of 95% confidence interval (Cis) and log rank *p* value.

### 2.4. TCGA and CBioPortal Analysis

The CBioPortal (http://www.cbiportal.org/) provides information of complex cancer genomics and clinical profiles from 105 cancers in TCGA database [16]. In this study, the genetic alteration, coexpression, and the network module of CXC chemokines were analyzed from CBioPortal. 606 EOC samples (TCGA, Firehose legacy) were analyzed. mRNA expression *z* scores (RNA Seq V2 RSEM) were obtained using a *z* score threshold of ±2.0. Protein expression *z* scores (mass spectrometry by CPTAC) were obtained using a *z* score threshold of ±2.0.

### 2.5. TIMER

TIMER (http://cistrom.shinyapps.io/timer/) is a database which can systematically describe infiltration of different immune cells along with certain genes and their clinic influence [17]. In our study, we used “Gene” module to evaluate correlation of CXC chemokines with infiltration of immune cells, including B cell, CD8+ T cell, CD4+ T cell, macrophage, neutrophil, and dendritic cell. “SCNA” module was used to compare immune infiltration levels in OC with different somatic copy number alterations.

### 2.6. Metascape

Metascape (http://metascape.org) is a free and reliable tool for gene annotation and enrichment analysis [18]. It is a useful database to analyze common and unique pathways within a group of targeted genes. In our study, Metascape was used to conduct pathway and analyze correlation neighbor genes with CXC chemokines.

### 2.7. LinkedOmics

LinkedOmics (http://www.linkedomics.org/) provides comprehensive multiomics data analysis across 32 TCGA cancer types [19]. “LinkInterpreter” module was used to evaluate biological value from kinase target enrichment, miRNA target enrichment, and transcription factor target enrichment of CXC chemokines. Gene Set Enrichment Analysis (GSEA) was performed to analyze a minimum number of genes (size) of 3 and a simulation of 500. Results were analyzed statistically using the Spearman correlation test. The *p* value cutoff was 0.05.

## 3. Results

### 3.1. CXC Chemokines Differentiated Expression in OC

**Figure 1:**
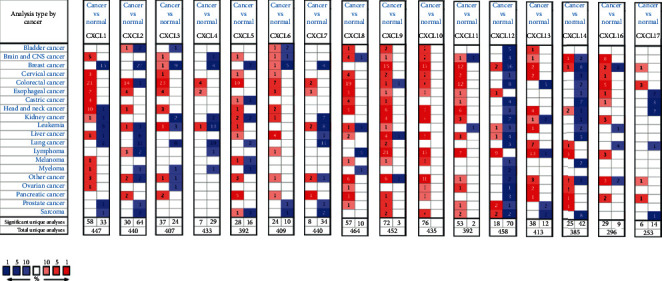
The mRNA levels of CXC chemokines in OC (ONCOMINE). The figure shows the number of datasets with high expression and low expression of CXC chemokines. Red color represents the over-expression datasets, while blue represents down-expression with statistical significance.

We first delved into the sixteen CXC chemokines' transcriptional expression levels in OC via ONCOMINE dataset. The results are shown in Figure 1 and Table 1. The transcriptional levels of CXCL1, CXCL8, CXCL10, CXCL11, CXCL12, CXCL13, and CXCL14 were significantly elevated while CXCL3 was obviously reduced in ovarian cancer vs normal ovarian tissue. These data were consistent with the research dataset of Welsh and Yoshihara who demonstrated that CXCL3 was remarkably decreased in ovarian serous adenocarcinoma or ovarian serous surface papillary adenocarcinoma compared with the normal tissue [20]. Adib et al. also found CXCL1 (*p*=0.004) in OC was increased with a fold change of 2.735 [21]. The research of Lu dataset showed that CXCL8 was elevated in ovarian mucinous adenocarcinoma compared with that in normal ovarian tissue [22]. Welsh and TCGA dataset both demonstrated that CXCL10 was obviously increased in EOC (fold change = 31.637, *p*=0.011, TCGA) [20,23]. The high expression levels of CXCL11 (fold change = 2.995, *p*=1.33*E* − 6, TCGA; fold change = 6.089, *p*=0.013, Yashihara ovarian), CXCL12 (fold change = 29.039, *p*=110*E* − 8, Yashihara ovarian), and CXCL13 (fold change = 2.901, *p*=2.94*E* − 8, TCGA; fold change = 2.107, *p*=0.001, Yashihara ovarian) in EOC were proved by Yashihara et al. and TCGA database [23]. The same results of CXCL13 were supported by Bonome dataset [24]. The fold change of CXCL14 was 2.762 (*p*=3.21*E* − 5), 3.372 (*p*=0.002), 2.683 (*p*=0.035), and 2.149 (*p*=6.37*E* − 6) in the dataset of Hendrix [25], Lu [22], and Bonome [24], respectively.

**Figure 2:**
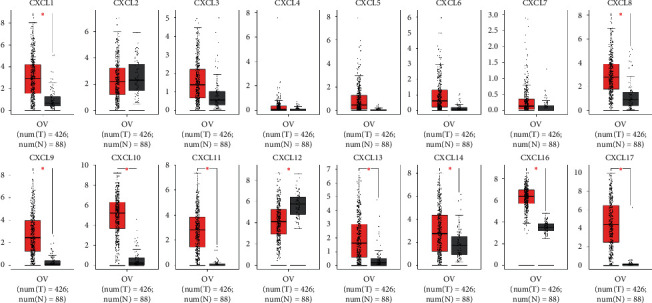
The expression of CXC chemokines in OC patients (GEPIA). Box plots derived from gene expression data for GEPIA comparing the expressions of CXC chemokines in ovarian cancer and normal ovarian tissue; the *p* value was set at 0.05. Results show that CXCL1, CXCL8, CXCL9, CXCL11, CXCL12, CXCL16, and CXCL17 were remarkably elevated in ovarian cancer compared to those in normal tissue. *∗*indicates that the results are statistically significant.

**Figure 3:**
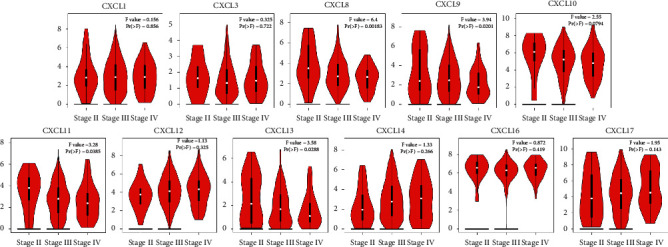
The correlation between differentially expressed CXC chemokines and the pathological stage of OC patients (GEPIA). ^*∗*^*p* < 0.05. Results show that CXCL8 (*p*=0.018), CXCL9 (*p*=0,020), CXCL11 (*p*=0.039), and CXCL13 (*p*=0.029) were highly correlated with clinic pathological stage.

We then assessed the transcriptional levels of CXC chemokines and their correlation with pathological stage of clinic in the database of GEPIA. Our results showed that CXCL1, CXCL8, CXCL9, CXCL11, CXCL12, CXCL16, and CXCL17 were remarkably elevated in ovarian cancer compared to those in normal tissue (Figure 2). Also, we found CXCL8 (*p*=0.018), CXCL9 (*p*=0.020), CXCL11 (*p*=0.039), and CXCL13 (*p*=0.029) were highly correlated with clinic pathological stages (Figure 3**)**. The expression of CXCL8, CXCL9, CXCL11, and CXCL13 was increased as the tumor stage increased. Our results suggested that CXC chemokines play a key role in the progression of ovarian cancer.

### 3.2. The Prognostic Value of CXC Chemokines in OC Patients

**Figure 4:**
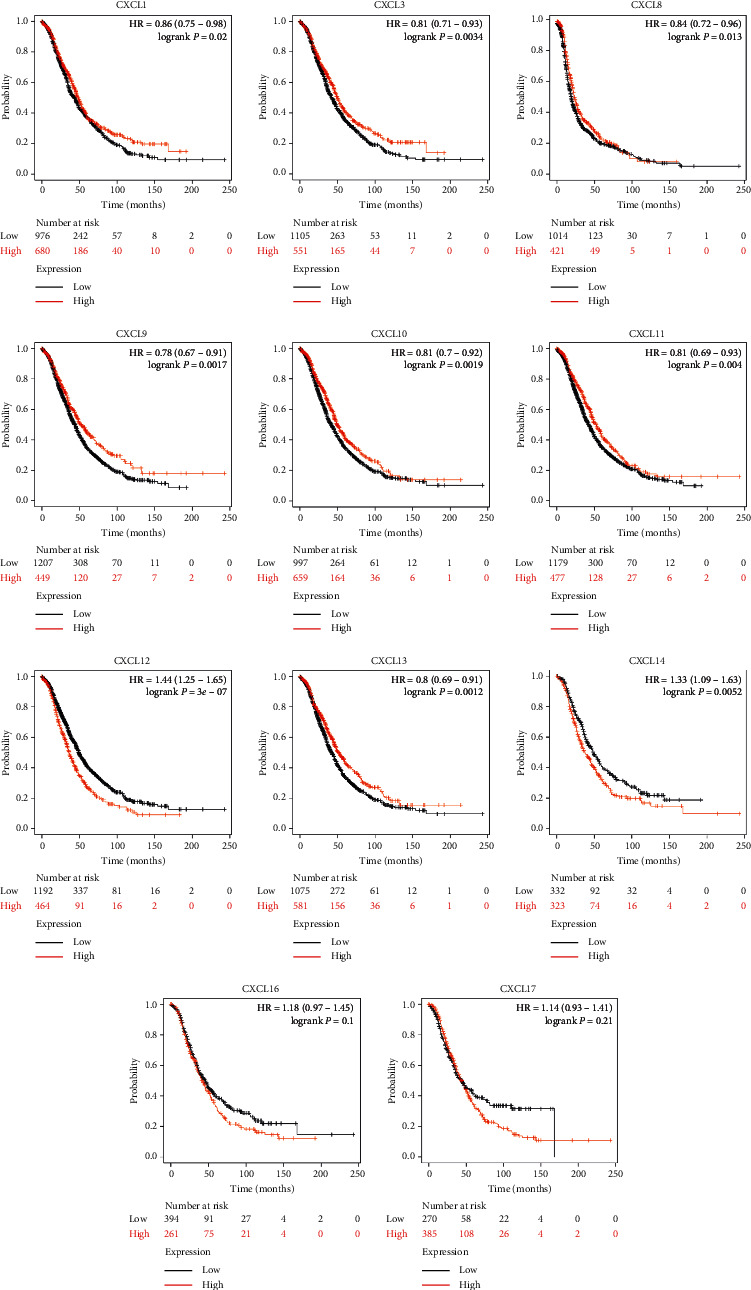
Kaplan–Meier curves reveal the overall survival (OS) differences based on mRNA level of CXC chemokines in OC patients. CXCL1, CXCL3, CXCL9, CXCL11, and CXCL13 (*p* < 0.05) are significantly correlated with long OS. Low expressions of CXCL12 and CXCL14 (*p* < 0.05) are associated with long OS.

**Figure 5:**
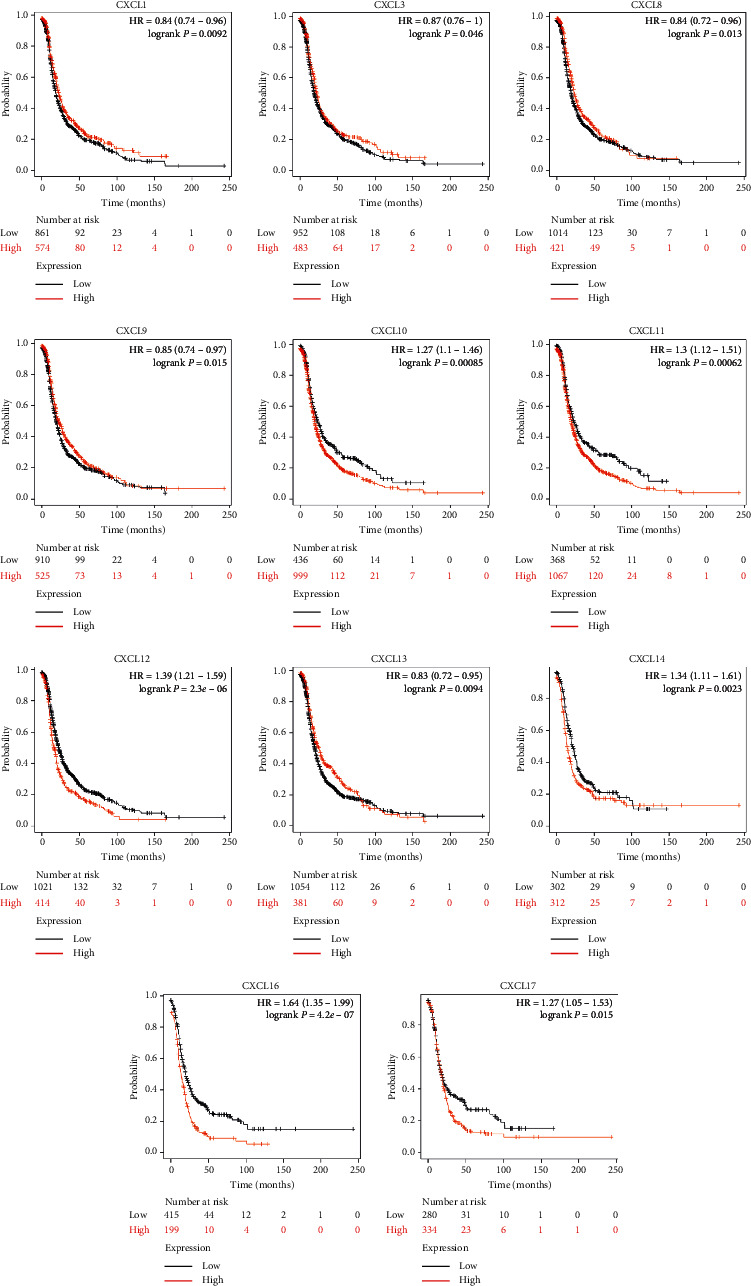
Kaplan–Meier curves reveal the progression-free survival (PFS) differences based on mRNA level of CXC chemokines in OC patients. High expression of CXCL1, CXCL3, and CXCL10 and low expression of CXCL11, CXCL12, CXCL14, CXCL16, and CXCL17 are remarkably correlated with long PFS.

**Figure 6:**
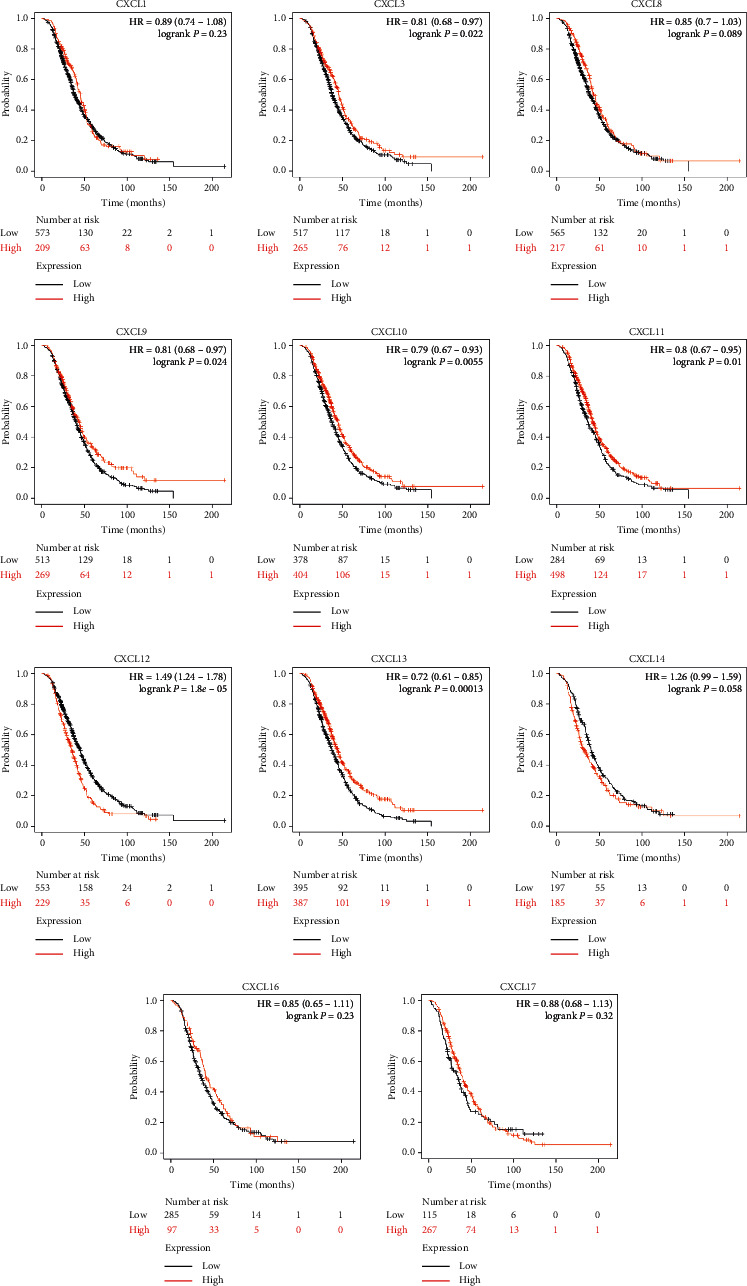
Kaplan–Meier curves reveal the post-progression survival (PPS) differences based on mRNA level of CXC chemokines in OC patients. High expression of CXCL3, CXCL9, CXCL10, CXCL12, and CXCL13 is correlated with long PPS.

We then analyzed the prognostic value of differentially expressed CXC chemokines in OC through the Kaplan–Meier plotter database. Overall survival (OS) curves are shown in Figure 4. Data suggested that OC patients with high expression of CXCL1 (*p*=0.02), CXCL3 (*p*=0.0034), CXCL9 (*p*=0.0017), CXCL11 (*p*=0.004), and CXCL13 (*p*=0.0012) were significantly correlated with long OS. However, low expression of CXCL12 (*p*=3*e* − 7) and CXCL14 (*p*=0.0052) was associated with long OS. The differential expressions of CXC chemokines of progression-free survival (PFS) and post-progression survival (PPS) were also assessed (Figures 5 and 6). We found that high expression of CXCL1, CXCL3, CXCL10, and low expression of CXCL11, CXCL12, CXCL14, CXCL16, and CXCL17 were remarkably correlated with long PFS, while high expression of CXCL3, CXCL9, CXCL10, CXCL12, and CXCL13 was shown with long PPS. Among these, CXCL13 (*p*=0.00013) was significantly correlated with long PPS. However, low expression of CXC12 (1.8*e* − 05) was relevant to long PPS. Our research suggested that CXCL3 and CXC12 could be used as potential prognostic markers of OC.

### 3.3. Gene Alteration, Coexpression, and Prognostic Value of Alterative CXC Chemokines in Patients with OC

**Figure 7:**
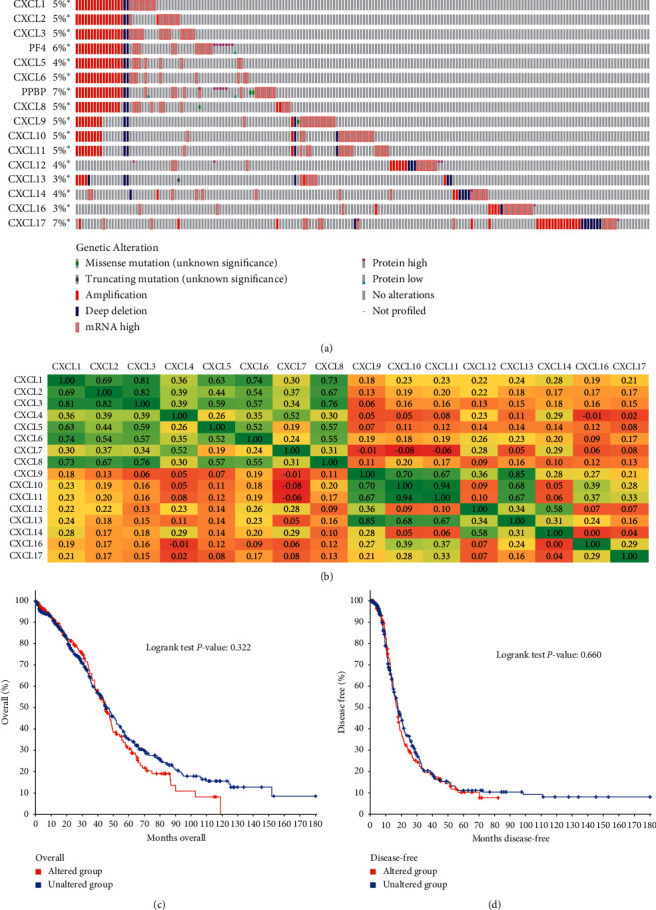
Genetic alteration, correlation of CXC chemokines, and prognostic value of altered CXC chemokines in OC. (a) Summary of alteration CXC chemokines in OC. (b) Correlation heatmap of CXC chemokines in OC. (c, d) The overall survival and disease-free survival analysis derived from CBioPortal database.

The TCGA and CBioPortal were used to analyze the gene alteration rate of CXC chemokines in OC. Among those differentially expressed CXC chemokines, our results showed that 5% of CXCL3, CXCL8, and CXCL11 were altered, while 4% of CXCL12 and CXCL14 were mutated. 3% of CXCL16 and 7% of CXCL17 suffered with gene alteration (Figure 7(a)). We then delved into the correlation of potential coexpression of CXC chemokines in OC; there was a moderate-to-high correlation among the expression of CXCL1, CXCL3, and CXCL8 within the differentiated genes, and a high correlation among CXCL9, CXCL10, and CXCL11 (Figure 7(b)), while a low correlation of CXCL12, CXCL13, CXCL14, CXCL16, and CXCL17 was detected (Figure 7(b)). We next explored the prognostic value of altered CXC chemokines in OC; we found there was no statistically significant correlation of overall survival and disease-free survival (Figures 7(c) and 7(d)).

### 3.4. Functional Enrichment Analysis of CXC Chemokines in OC Patients

**Figure 8:**
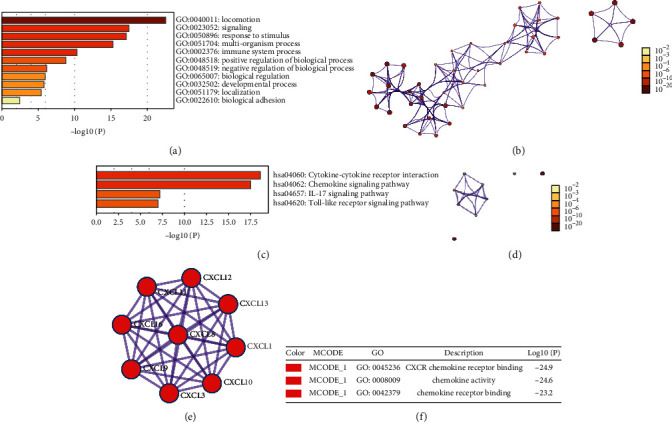
Enrichment analysis of CXC chemokines and neighboring genes in OC (Metascape). (a) Heatmap of Gene Oncology (GO) enriched terms colored by *p* value. (b) Network of GO enriched terms colored by *p*-value. (c) Heatmap of Kyoto Encyclopedia of Genes and Genomes (KEGG) enriched terms colored by *p* value. (d) Network of KEGG enriched terms colored by *p*-value. (e) Protein-protein interaction (PPI) network and the most significant MCODE from the PPI network. (f) Functional analysis of MCODE1 components.

GEPIA and Metascape were utilized to analyze the functions, pathway enrichment, and their neighboring genes of differentially expressed CXC chemokines in OC (Figure 8). We delved into the top 10 associated genes of each differentiated CXC chemokine via GEPIA dataset (Table 2). The top 11 GO enrichment items of those differentiated CXC chemokines focused on locomotion, signaling, and response to stimulus. Also, they undergo the process of multiorganism, immune system, biological regulation, etc (Figures 8(a) and 8(b) and Table 3). KEGG pathway enrichment analysis represented that cytokine-cytokine receptor interaction, chemokine signaling pathway, IL-17 signaling pathway, and toll-like receptor signaling pathway were significantly involved in the tumorigenesis and pathogenesis of OC (Figures 8(c) and 8(d) and Table 4). Moreover, to better understand the relationship between CXC chemokine family members and OC, we then performed a Metascape protein-protein interaction (PPI) enrichment analysis. The PPI network and MCODE components are shown in Figures 8(e) and 8(f). Data showed that the biological functions of CXC chemokines are mainly enriched in CXCR chemokine receptor binding, chemokine activity, and chemokine receptor binding in OC.

### 3.5. Immune Cell Infiltration of CXC Chemokines in OC Patients

**Figure 9:**
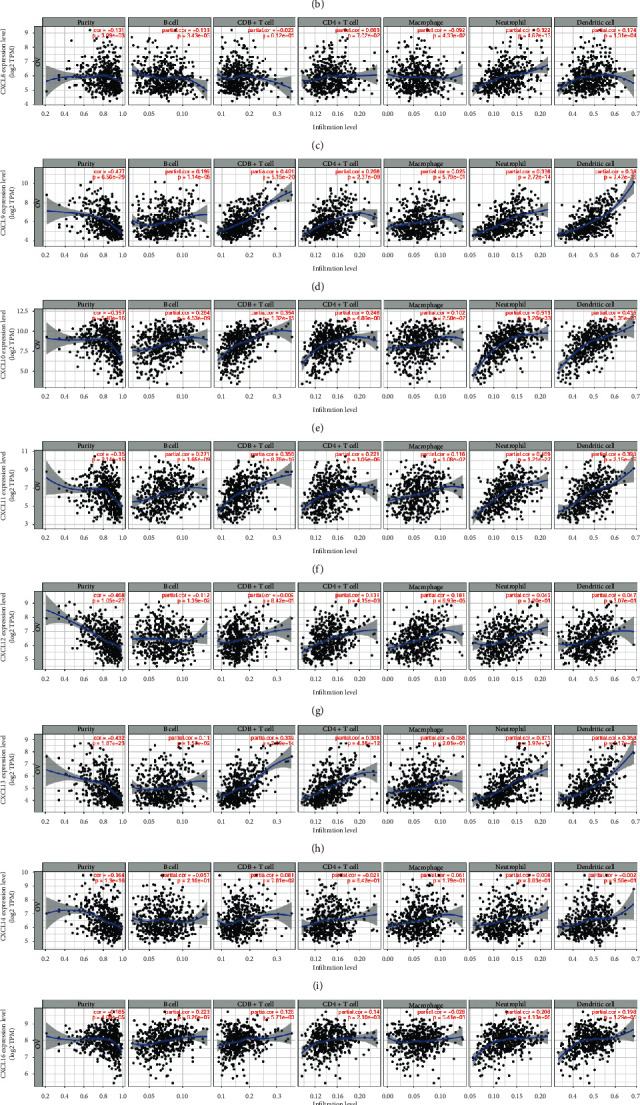
The correlation of differentially expressed CXC chemokines and immune cell infiltration (TIMER).The correlation between the immune cells and expression of (a) CXCL1, (b) CXCL3, (c) CXCL8, (d) CXCL9, (e) CXCL10, (f) CXCL11, (g) CXCL12, (h) CXCL13, (i) CXCL14, (j) CXCL16, and (k) CXCL17 in OC.

**Figure 10:**
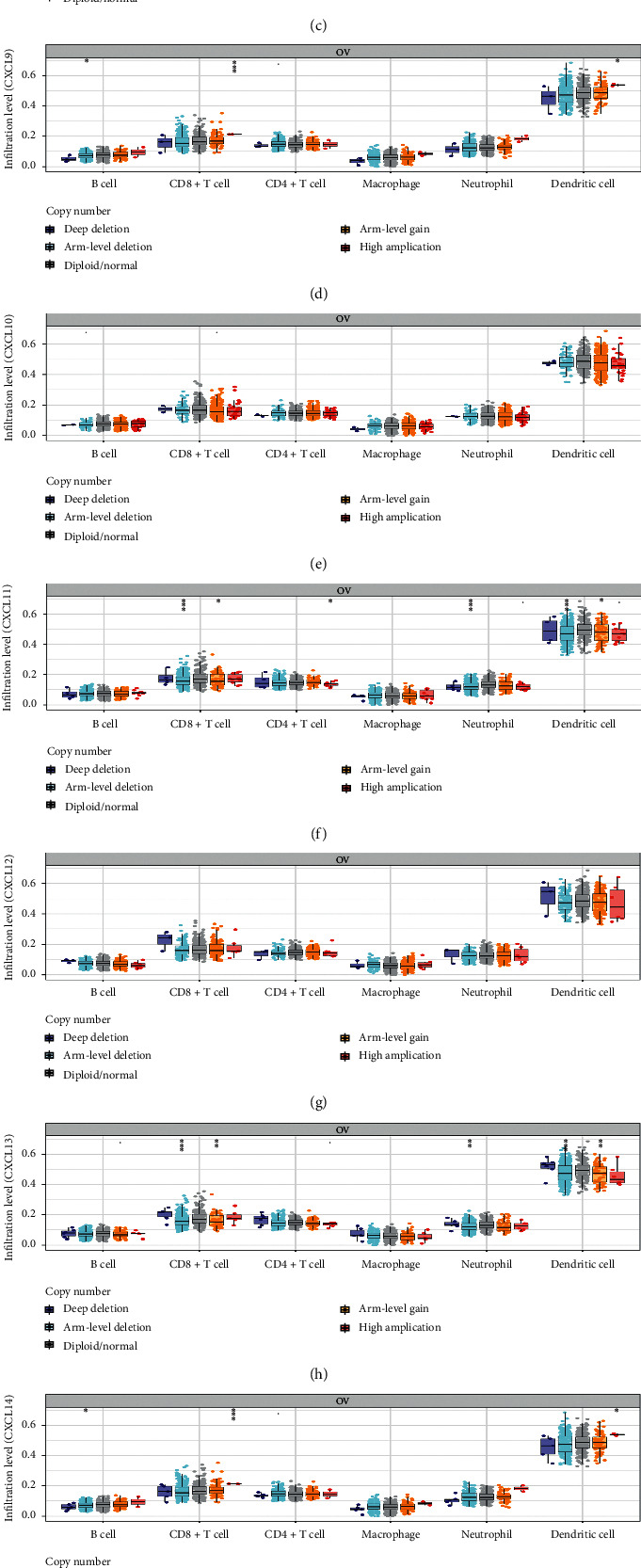
The infiltration of immune cells caused by gene copy number alteration of differentially expressed CXC chemokines (TIMER).

Immune cells are the main cells of TME and infiltration of immune cells plays a pivotal role in tumor progression. Therefore, we further explored the correlation of differentially expressed CXC chemokines and immune cells infiltration using TIMER database. Among these chemokines, we found that B cells' infiltration was negatively correlated with CXCL1, CXCL3, CXCL8, CXCL12, and CXCL14, while their infiltration was positively correlated with CXCL9, CXCL10, CXCL11, CXCL13, CXCL16, and CXCL17 (Figures 9(a)–9(k)). CD8+ T cells had a negative correlation with CXCL1, CXCL3, CXCL8, and CXCL12, while they had a positive correlation with CXCL9, CXCL10, CXCL11, CXCL13, CXCL14, CXCL16, and CXCL17 (Figures 9(a)–9(k)). CD4+ T cells' infiltration existed in almost all differentiated CXC chemokines, except for CXCL14 (Cor = 0.021, *p*=6.42*e* − 01) (Figure 9(i)). The level of macrophages infiltration was negatively associated with CXCL1, CXCL3, CXCL8, and CXCL16, while it was positively associated with CXCL9, CXCL10, CXCL11, CXCL12, CXCL13, CXCL14, and CXCL17 (Figures 9(a)–9(k)). The infiltration of neutrophils was all positively correlated with differentiated CXC chemokines in OC (Figures 9(a)–9(k)). CXCL3 (Cor = −0.025, *p*=5.82*e* − 01) and CXCL14 (Cor = 0.002, *p*=9.5*e* − 01) were negatively associated with the infiltration of dendritic cells (Figures 9(b) and 9(i)). Interestingly, we found that CXCL9, CXCL10, CXCL11, CXCL13, and CXCL17 all suffered with high infiltration of immune cells (Figures 9(d)–9(f), 9(h), and 9(k)). The module “SCAN” of TIMER was used to delve into the infiltration of immune cells caused by gene copy number alteration of differentiated CXC chemokines. Our results proved that the alteration of gene copy number, to some extent, could influence the infiltration of immune cells (Figure 10).

### 3.6. Kinase Targets and mRNA Targets of CXC Chemokines in Patients of OC

We then analyzed the kinase targets and mRNA targets of differentially expressed CXC chemokines from LinkedOmics database (Table 5). PLK1 and ATM were the top two targets in the CXCL1 kinase target network. The targets of CXCL3 were ATM and CDK2. LCK, ATM and ATR, ATM were the top two kinase targets in the CXCL8 and CXCL14, respectively. LCK and FYN were the targets of CXCL9 and CXCL13. LCK and LYN were the top two targets of CXCL10, CXCL11, CXCL12, CXCL16, and CXCL17 kinase target networks. Similarly, we explored the enriched mRNA targets from LinkedOmics database (results presented in Table 6). The top two enriched mRNA targets were ACTGCCT, MIR-34B and CACCAGC, MIR-138 in CXCL1. CCAGGTT, MIR-490 and CCCAGAG, MIR-326 were mainly enriched in CXCL3. As the table describes, GACAGGG, MIR-339 and CCCAGAG, MIR-326 were enriched in CXCL8, while GGTGTGT, MIR-329 and ACAGGGT, MIR-10A, MIR-10B were enriched in CXCL9. The enriched mRNA targets of CXCL10, CXCL11, CXCL12, CXCL13, CXCL14, CXCL16, and CXCL17 are elaborated in Table 6.

## 4. Discussion

CXC chemokines are primarily identified as the mediator in the inflammatory milieu; they are responsible for the recruitment and activation of immune cells. In addition, they mediate pro- and antiangiogenic effects. Recently, researchers have demonstrated that chemokines can affect progression of several tumors such as lung cancer, bladder cancer, prostate cancer, and ovarian cancer through several aspects, including angiogenesis, proliferation, migration, invasion, and metastasis [6–8, 26].

As CXC chemokines and their receptors may be potentially used in molecular targeting of cancers, accumulating researches have focused on CXC chemokine family and their receptors in ovarian cancer. For instance, researchers found that tumor-suppressor miRNA-27b-5p regulated the growth and metastatic behaviors of ovarian carcinoma cells by targeting CXCL1 [27]. CXCL4 insufficiency was involved in specific inflammatory microenvironment of ovarian cancers arising in endometriosis [28]. Recombinant CXCL8 (rIL-8) attenuated si-JMJD2A-suppressed malignancy of OC cells(OCC) and CXCL8 induced proliferation of OCC in 3D Spheroids [29, 30]. In a mouse model of high-grade serous ovarian cancer, CXCL10 altered the tumor immune microenvironment and facilitated disease progression [31]. Researchers also found that upregulated CXCL14 and CXCL16 were associated with poor survival outcomes and promoted ovarian cancer cells proliferation [32, 33]. In the progression of OC, higher CXCL17 correlated with higher expression of B7–H4 [34]. All these researches showed the importance of CXC chemokines in the progression of OC. However, the patterns of expression and exact roles of CXC chemokine family in OC are obscure. In our study, we systematically explored the expression patterns, prognostic values, gene alteration, coexpressions, correlation, infiltration of immune cells, potential functions, pathways, and kinase/mRNA targets of differentially expressed CXC chemokines in OC.

We firstly explored the expression of CXC chemokines and their correlation with pathological stage in OC. We found that ten CXC chemokines (CXCL1/8/9/10/11/12/13/14/16/17) were highly expressed, while CXC3 was reduced in OC. These results were in accordance with previous researches; CXCL14 and CXCLL16 were highly expressed in OC and they were associated with poor survival outcomes and promoted OCC proliferation [32, 33]. Also, we found that CXCL8/9/11 and CXCL13 were highly correlated with clinic pathological stage. Patients with low expression of CXCL8/9/11/13 were associated with better prognosis. Moreover, we utilized the Kaplan–Meier plotter to find the potential prognostic markers of OC. We found patients with high expression of CXCL3 and CXCL12 had a significantly better prognosis. Herein, CXCL3 and CXCL12 may be used as novel prognostic markers of OC in the future.

Then, we analyzed the genetic alterations and coexpression of differentially expressed CXC chemokines via TCGA and CBioPortal database. The differentially expressed CXC chemokines all contained a certain proportion of genetic alteration while the altered genes did not affect the prognosis of OC. We found a low-to-high correlation among these differentially expressed genes; it suggested that these CXC chemokines may play a synergistic role in OC progression.

Furthermore, we used GO and KEGG pathway enrichment analysis to identify the main functions and pathways of differentially expressed CXC chemokines in OC. Their functions focused on locomotion, signaling, response to stimulus, undergoing the process of multiorganism, immune system, biological regulation, etc. The cytokine-cytokine receptor interaction, chemokine signaling pathway, IL-17 signaling pathway, and toll-like receptor signaling pathway were the vital pathways that participated in OC. For instance, researchers found that cancer-associated fibroblasts (CAFs) in TME induced epithelial-mesenchymal transition (EMT) and cisplatin resistance in OC via CXCL12/CXCR4 axis [35]. In breast cancer, IL-17-CXCR2 axis facilitated the recruitment of neutrophils to the tumor sites, thus allowing them to play a cancer-promoting role in cancer progression. [36]. We then investigated the kinase targets of differentially expressed CXC chemokines; the kinase targets were mainly in ATM, FYN, LYN, LCK, PLK1, CDK2, and ATR. These results may provide us with potential therapeutic targets in OC.

Recently emerging data suggested that immune cell infiltration plays a key role in tumor progression [37]. Our research delved into infiltration of six immune cells (B cells, CD8^+^ T cells, CD4^+^ T cells, macrophages, neutrophils, and dendritic cells) that were correlated with differentiated CXC chemokines. The results indicated that CXC chemokines could be immune regulators in OC.

All in all, our research took advantages of public databases to systematically delve into data of CXC chemokines in OC. However, our results may need to be verified by in-depth experiments in vivo and in vitro. Multiple clinical trials are needed to validate potential biomarkers of CXC chemokines. We hope our results can provide novel insights for our researchers and these potential targets could be applied in clinic someday.

## Figures and Tables

**Table 1:** The transcription levels of CXC chemokines in different types of OC and normal ovarian tissues (ONCOMINE).

TLR	Type of ovarian cancer versus normal ovarian tissue	Fold change	*p* value	*t*-test	References	PMID
CXCL1	Ovarian serous adenocarcinoma vs. normal	2.753	0.004	4.093	Adib ovarian	14760385
CXCL3	Ovarian serous surface papillary carcinoma vs. normal	−5.279	1.57*E* − 5	−4.912	Welsh ovarian	11158614
	Ovarian serous adenocarcinoma vs. normal	−7.691	0.004	−3.179	Yoshihara ovarian	19486012
CXCL8	Ovarian mucinous adenocarcinoma vs. normal	2.455	0.015	2.562	Lu ovarian	15161682
CXCL10	Ovarian serous adenocarcinoma vs. normal	9.290	2.38*E* − 5	5.697	Yoshihara ovarian	19486012
	Ovarian serous surface papillary carcinoma vs. normal	31.673	0.011	3.620	Welsh ovarian	11158614
	Ovarian serous cystadenocarcinoma vs. normal	7.751	4.65*E* − 4	5.346	TCGA ovarian	
CXCL11	Ovarian serous cystadenocarcinoma vs. normal	2.995	1.33*E* − 6	9.548	TCGA ovarian	
	Ovarian serous adenocarcinoma vs. normal	6.089	0.013	2.918	Yoshihara ovarian	19486012
CXCL12	Ovarian serous adenocarcinoma vs. normal	29.039	1.10*E* − 8	−13.783	Yoshihara ovarian	19486012
CXCL13	Ovarian carcinoma vs. normal	2.245	2.66*E* − 23	12.237	Bonome ovarian	18593951
	Ovarian serous cystadenocarcinoma vs. normal	2.091	2.94*E* − 8	10.921	TCGA ovarian	
	Ovarian serous adenocarcinoma vs. normal	2.107	0.001	3.226	Yoshihara ovarian	19486012
CXCL14	Ovarian clear cell adenocarcinoma vs. normal	2.762	3.21*E* − 5	6.752	Hendrix ovarian	16452189
	Ovarian clear cell adenocarcinoma vs. normal	3.372	0.002	4.385	Lu ovarian	15161682
	Ovarian endometrioid adenocarcinoma vs. normal	2.683	0.035	2.097	Lu ovarian	15161682
	Ovarian carcinoma vs. normal	2.149	6.37*E* − 6	5.915	Bonome ovarian	18593951

**Table 2:** The top 10 significant genes correlated with differentially expressed CXC chemokines in OC.

IRFs	Correlated genes
CXCL1	IL8, ADM, CXCL3,CXCL6, TTC9, CXCL5, NDUFA4L2, PTGS2, CCL20, CACNA1B
CXCL3	CXCL2, IL8, ZC3H12A, CXCL1, NFKBIZ, NFKBIA, TNF, CEBPD, IER3, CCL20
CXCL8	CXCL1, CXCL3, BCL2A1, CXCL5, TREM1, CXCL6, ZC3H12A, CCL20, NFKBIZ, CXCL2, IL1RN
CXCL9	CXCL13, CD3D, SIRPG, CXCR6, SLAMF7, CD2, TIGIT, SLAMF6, CD8A, CD3G, IGJ
CXCL10	CXCL11, TAP1, LOC400759, PSMB9, CD38, HLA-C, HLA-F, CXCR2P1, B2M, UBE2L6,HLA-A
CXCL11	CXCL10, LOC400759, TAP1, PSMB9, HLA-F, TNFSF13B, TRIM22, ETV7, BATF2, LAG3
CXCL12	CNRIP1, HIC1, ZEB2, PDGFRA, ANTXR2, DCN, TGFB1I1, ZCCHC24, FAM198B, PTGIS
CXCL13	CXCL9, CD3D, SIRPG, PDCD1, CXCR6, TIGIT, CD2, SLAMF6, IGJ, CD3G
CXCL14	SFRP2, FBN1, LUM, DCN, MATN3, C1QTNF3, ISM1, MMP2, COPZ2, FAP
CXCL16	ZMYND15, MINK1, ARRB2, TAPBP, ME2, NFKB1, MYD88, CTSS, GPRIN3, CLDN7, APOB48R
CXCL17	RARRES3, CEACAM1, MESP1, PSMB10, FUT2, SNORA74B, SRD5A3, SQRDL, SECTM1, LRRC26, HLA-C

**Table 3:** GO enrichment items of those differentiated CXC chemokines (Metascape).

GO	Category	Description	Count	(%)	Log10 (P)	Log10 (q)
GO:0008009	GO molecular functions	Chemokine activity	10	90.91	−26.35	−22.00
GO:0045236	GO molecular functions	CXCR chemokine receptor binding	8	72.73	−23.63	−19.76
GO:0030595	GO biological processes	Leukocyte chemotaxis	11	100.00	−22.53	−18.78
GO:0048248	GO molecular functions	CXCR3 chemokine receptor binding	4	36.36	−12.95	−9.98
GO:0002690	GO biological processes	Positive regulation of leukocyte chemotaxis	6	54.55	−12.04	−9.09

**Table 4:** KEGG enrichment items of those differentiated CXC chemokines (Metascape).

GO	Category	Description	Count	%	Log10 (P)	Log10 (q)
Hsa04060	KEGG pathway	Cytokine-cytokine receptor interaction	10	90.91	−18.59	−15.90
Hsa04062	KEGG pathway	Chemokine signaling pathway	9	81.82	−17.49	−15.10
Hsa04657	KEGG pathway	IL-17 signaling pathway	4	36.36	−7.19	−4.98
Hsa04620	KEGG pathway	Toll-like receptor signaling pathway	4	36.36	−7.00	−4.90

**Table 5:** The kinase target networks of CXC chemokines in OC (LinkedOmics).

CXC chemokines	Enriched kinases target	Description	Leading EdgeNum	*p*-value
CXCL1	Kinase_PLK1	Polo-like kinase 1	33	0
	Kinase_ATM	ATM serine/threonine kinase	68	0
CXCL3	Kinase_ATM	ATM serine/threonine kinase	52	0
	Kinase_CDK2	Cyclin-dependent kinase 2	106	0
CXCL8	Kinase_LCK	LCK proto-oncogene, Src family tyrosine kinase	20	0
	Kinase_ATM	ATM serine/threonine kinase	40	0
CXCL9	Kinase_LCK	LCK proto-oncogene, Src family tyrosine kinase	17	0
	Kinase_FYN	FYN proto-oncogene, Src family tyrosine kinase	14	0
CXCL10	Kinase_LCK	LCK proto-oncogene, Src family tyrosine kinase	23	0
	Kinase_LYN	LYN proto-oncogene, Src family tyrosine kinase	17	0
CXCL11	Kinase_LCK	LCK proto-oncogene, Src family tyrosine kinase	21	0
	Kinase_LYN	LYN proto-oncogene, Src family tyrosine kinase	18	0
CXCL12	Kinase_LYN	LYN proto-oncogene, Src family tyrosine kinase	19	0
	Kinase_LCK	LCK proto-oncogene, Src family tyrosine kinase	21	0
CXCL13	Kinase_LCK	LCK proto-oncogene, Src family tyrosine kinase	21	0
	Kinase_FYN	FYN proto-oncogene, Src family tyrosine kinase	14	0
CXCL14	Kinase_ATR	ATR serine/threonine kinase	39	0
	Kinase_ATM	ATM serine/threonine kinase	44	0
CXCL16	Kinase_LCK	LCK proto-oncogene, Src family tyrosine kinase	27	0
	Kinase_LYN	LYN proto-oncogene, Src family tyrosine kinase	24	0
CXCL17	Kinase_LCK	LCK proto-oncogene, Src family tyrosine kinase	20	0
	Kinase_LYN	LYN proto-oncogene, Src family tyrosine kinase	21	0

**Table 6:** The miRNA target networks of CXC chemokines in OC (LinkedOmics).

CXC chemokines	Enriched miRNA Target	Leading EdgeNum	*p*-value
CXCL1	ACTGCCT, MIR-34B	35	0
	CACCAGC, MIR-138	35	0
CXCL3	CCAGGTT, MIR-490	22	0
	CCCAGAG, MIR-326	50	0
CXCL8	GACAGGG, MIR-339	61	0
	CCCAGAG, MIR-326	141	0
CXCL9	GGTGTGT, MIR-329	27	0
	ACAGGGT, MIR-10A, MIR-10B	40	0
CXCL10	CGCTGCT, MIR-503	12	0
	GTCTTCC, MIR-7	51	0
CXCL11	GTGTGAG, MIR-342	22	0
	CCCAGAG, MIR-326	58	0
CXCL12	AGTCTAG, MIR-151	4	0
	ACATATC, MIR-190	14	0
CXCL13	GTGTGAG, MIR-342	24	0
	CGCTGCT, MIR-503	11	0
CXCL14	AGTCTAG, MIR-151	4	0
	AATGTGA, MIR-23A, MIR-23B	76	0
CXCL16	TGGTGCT, MIR-29A, MIR-29B, MIR-29C	133	0
	CAGCCTC, MIR-485-5P	38	0
CXCL17	GTATTAT, MIR-369-3P	62	0
	TGGTGCT, MIR-29A, MIR-29B, MIR-29C	147	0

## Data Availability

All data were acquired from public databases, including ONCOMINE, GEPIA, Kaplan–Meier plotter, cBioPortal, TIMER, Metascape, and LinkedOmics.
